# Revisiting discourse analysis in medical education research

**DOI:** 10.5116/ijme.6278.c1b7

**Published:** 2022-05-30

**Authors:** Rintaro Imafuku, Takuya Saiki, Robyn Woodward-Kron

**Affiliations:** 1 Medical Education Development Center, Gifu University, Japan; 2 Department of Medical Education, The University of Melbourne, Australia

**Keywords:** Discourse analysis, medical education research, learning interactions, communication studies

## Introduction

Viewed broadly, discourse analysis is the study of ways in which naturally occurring language is used between people, both in written and spoken contexts. In a learning context, discourse analysis of written and spoken texts is useful for making visible how educators, learners, and others engage in an educational activity to achieve learning goals.^1^ Examining how meaning is negotiated and constructed through language and interactions in learning contexts is a field of enquiry with a long history in the humanities and social sciences. Medical education researchers increasingly recognise the potential of discourse analysis to explain salient as well as underlying patterns and dynamics in learning interactions pertaining to clinical and social contexts.^2^ Close examination of interactional processes in settings, such as problem-based learning, communication training, feedback conversations, interprofessional practice, and the debriefing process in simulation-based education, may yield more multi-faceted evidence and afford valuable insights into teaching and learning.^3^ Despite its potential as an analytical tool in health professions education research, its application is still quite limited.

By reviewing and discussing a sample of discourse-oriented studies in medical education, this article aims to revisit and argue for the relevance of discourse analysis approaches for medical education research. We also aim to highlight how such discourse-oriented research can inform teaching and learning in medicine.

## Discourse analysis approaches and key concepts

Discourse analysis is a methodological approach to describe people's lived experiences through interaction from a language-in-use perspective, such as what the written or spoken text is about, who is involved, and how the text is organised and functions in a specific context, such as a problem-based learning classroom, or a bedside tutorial.^4^ Specifically, it is concerned with language use beyond the boundaries of a sentence, interrelationships between language and society and the interactive properties of everyday communication, which is different from a conversation analytical approach that focuses on speakers' social action itself through language.^5^

Discourse-oriented studies range from a macro-level analysis of societal institutions (e.g. curriculum documents in policies for selection), meso-level analysis of discourse practice (e.g. verbal behaviours and learning in a context of PBL classroom) to micro-level analysis of texts in a specific context (e.g. patient-provider interactions), influenced by various disciplines, including anthropology, cultural studies, linguistics, psychology, and sociology.^6^ Depending on the aims and foci of the studies, different types of written and spoken data are collected, and different approaches to discourse analysis are adopted. We overview the approaches from three perspectives: power in society (macro), social context and discourse practice (meso), and discourse structure and function (micro).

### Macro-level: discourse and power in society

A macro-level analysis of discourse is a critical discourse analysis focusing on relationships between language, ideology, and power. Here, language is seen as a form of social practice; the focus is on studying the way social power abuse, dominance, and inequality are enacted, reproduced, and resisted by text and talk in the social and political context.^7^ Accordingly, it explores why conversation occurs in terms of cultural, political, and organisational dimensions of the social world.^8^^,^^9^

Critical discourse analysis is increasingly employed as a research methodology in medical education. For example, it has been used to identify dominant ultrasound discourses,^10^ and utilised in the contexts of interprofessional collaboration and learning systems,^11^^,^^12^ professional identity of clinical students in cross-cultural settings,^13^and accreditation standards.^14^^,^^15^ This approach mainly analyses written texts, interviews, and narrative data to identify discourses that are important to particular social constructions and to uncover power relations between discourses.^9^

Specifically, we elaborate on one study to show how critical discourse analysis has been applied and the new knowledge is identified. Chen and colleagues conducted a critical discourse analysis of compassionate care within texts of undergraduate medical education accreditation standards in North America in effect since 1957.^15^ The detailed analysis of the text revealed a historically relative absence of language pertaining to compassionate care. It also identified the dominant discourses related to competencies and outcomes emphasised in the standards. The findings have shown how the construct of compassionate care can be incorporated into accreditation standards, which are essential and powerful texts in medical education systems.^15^

Further, these researchers have provided accessible analytical steps to undertaking Foucauldian critical discourse analysis; that is, (i) a familiarisation phase; (ii) assembly of an appropriately comprehensive archive of texts; (iii) analysis of the assembled archive to identify prominent keywords and statements; (iv) analysis of links between the identified dominant discourses and the values of compassionate care, and (v) description of the effects and implications of the dominant discourses on the potential to advance educational practices related to compassionate care.^14^^,^^15^ Breaking down the analytical stages of critical discourse analysis in this way provides an invaluable guide to novice medical education researchers.

### Meso-level: social context and language-in-use

Context is a key conception in the analysis of discourse practice. For example, ethnography of speaking is concerned with understanding the social contexts of linguistic interactions.^16^ A speech event is the prime unit of analysis. Its components are listed in a 'SPEAKING' grid, where S stands for setting or scene, P for the participant, E for ends, A for act sequence, K for key, I for instrumentalities, and N for norms of interaction and interpretation, and G for the Genre. This grid provides a reminder of the contextual dimensions in communicative events.^17^

Similarly, interactional sociolinguistics, which evolved through Gumperz's linguistics and anthropology^18^ and Goffman's sociology,^19^ also emphasises the importance of context in the interpretation of discourse. Notably, in this approach, Goffman developed frame analysis. The term 'frame' refers to basic cognitive structures which guide how we perceive and represent reality^;19^ its notion is to capture what people think they are doing when they talk to each other. Hence, frame analysis is a useful approach which explores different idea elements between people in a communicative event. For example, Sundberg, Reeves, Josephson and Nordquist adopted frame analysis to explore the meanings of interprofessional education by comparing educational leaders' perceptions with educational policy documents.^20^ They found differences regarding the definition, rationale, and presentation of 'interprofessional education' between the frames of documents and educators. The interprofessional education frame of educational leaders implied difficulties regarding the implementation of interprofessional education.^20^ 

The other primary aim of the meso-level analysis of discourse practice is to reveal the underlying meaning of a text and communicative behaviour. Speech act theory and pragmatics focus on the interpretation of utterances in discourse and the relation of speech to action during the conversation. Speech act theory attempts to conceptualise the speaker's intent to achieve a particular purpose through a linguistic lens. Austin argues that the performance of a statement which delivers textual information is a certain kind of action in and of itself and that a single utterance can produce three kinds of acts concomitantly.^21^ That is, a locutionary act is, roughly, the utterance of a given statement with given sense and references. An illocutionary act performed in the utterance involves its conventional force or implied meaning, such as informing, ordering, warning, and so forth. A perlocutionary act is performed when we say something to achieve a goal, such as by convincing, persuading, deterring, and surprising.^22^ Searle classifies illocutionary speech acts into five types: assertives, directives, commissives, expressives, and declaratives.^22^ This classification helps researchers to specify the type of verbal behaviours in social contexts. For example, Manalastas, Noble, Viney and Griffin examined the functions of the verbal behaviours of doctors while consulting a patient.^23^ Specifically, using the framework of speech acts, they characterised the pragmatic meaning of the signalling behaviour within the doctor's utterances, which may be important in facilitating or inhibiting patient autonomy.^23^

### Micro-level: discourse structure and function

A micro-level analysis of discourse explores how text is linguistically and functionally organised in specific communicative contexts. In other words, this approach aims to uncover the structural-functional aspects of discourse. Specifically, the Birmingham School of Linguistics and Systemic Functional Linguistics are major schools of this approach and aim to describe the structure of written and spoken texts and relate them to social contexts and other linguistic features.

Regarding the discourse structure, Sinclair and Coulthard from the Birmingham School systematically describe discourse units in a pedagogic context.^24^ Specifically, acts make up moves, which in turn make up exchanges, which make up transactions, which, finally, make up lessons, the largest discourse unit. The Birmingham School's basic unit of conversation structure is an exchange comprising three moves: initiation, response, and feedback/follow-up sequence.^24^^,^^25^ That is, a teacher initiates the exchange with a question, the pupil responds, and the teacher provides feedback. This sequence is a framework for characterising a pattern of classroom interaction.

As to discourse function, Systemic Functional Linguistics views language as a resource for making multiple strands of meaning simultaneously, including representational meanings about the world and experiences, interpersonal meanings about roles and relationships, and textual meanings about the message.^26^ It enables researchers to explore how language selected by a speaker or writer is functional for constructing meanings.^26^^,^^27^

Genre theory, developed by systemic functional linguists particularly interested in school education, is an accessible approach to discourse analysis for medical education researchers.^28^^, ^^29^ Genres can be characterised as recurring text types with recognisable language features and stages that contribute to the text achieving its social purpose such as a research presentation.^4^^,^^29^ It offers a framework for analysing how text is organised and sequenced across communicative events.^17^ An analytical procedure for genre analysis is as follows: (i) identify the overall purpose or goal of the Genre; (ii) identify the predictable main stages and substages in the text; (iii) provide a rationale for delineating between stages; (iv) identify the order of the stages and classify stages as optional or are recursive; (v) provide functional labels to the stages and substages to describe their contribution to the Genre; (vi) identify similarities and differences in the organisation and the logic behind the arrangement of information, if comparing to other text in the same Genre. Delineating texts in this way can assist with identifying differences between effective and ineffective learner communication such as in handovers when key stages are missing or not sufficiently prioritised.^30^

For example, taking a structural-functional discourse analysis approach, two of the present authors and their colleagues investigate interactions among interdisciplinary groups of medical, dentistry, pharmaceutical sciences, nursing occupational therapy, and physiotherapy students working together in problem-based learning tutorials.^31^ Discourse analysis in that study indicates two distinctive interaction patterns of knowledge building in interprofessional problem-based learning tutorials: co-construction of knowledge among students from different disciplines and elaboration of knowledge between students from the same discipline.^31^

### Micro-level: interactional research into medical education

In addition to discourse structure and function, studies on interactions using a more fine-grained perspective are gradually becoming more established in medical education research. This approach to discourse analysis aims to identify the distinctive interaction patterns resulting from negotiation of meaning-making in the phenomenon investigated. In a few these studies from this perspective, the research contexts are generally classified into communicative events with multiple participants or dyadic encounters.^4^

Learning encounters among multiple participants in medical education has been a rich source of interest to the discourse analyst with an educational lens, for example, verbal interactions in group learning settings in the context of problem-based learning,^32^^-^^35^ interprofessional education,^36^ case-based learning,^37^ and group reflection sessions.^38^ In turn, discourse-oriented studies on dyadic encounters include roleplay activities in medical student/physician-simulated patient communication,^23^^,^^39^^,^^40^ communicative events in OSCEs,^41^^,^^42^ doctor-patient consultation in emergency department,^43^ and feedback interaction in clinical education settings.^44^ For example, regarding dyadic encounters, Pun explored how Chinese medical students communicate bad news to simulated patients in roleplay sessions during communication training.^40^ Pun applied an ethnographic discourse approach to analyse the videotaped verbal interactions, revealing that students displayed six discourse strategies regarding patient-oriented communicative patterns to cater to patients' emotional and practical needs. Pun's work highlighted the complexity of language used by medical professionals when disclosing bad news to patients within a specific cultural context.^40^ Therefore, interactional research into medical education provides researchers with an emic perspective of the participants, which could be foundational for educational development.

Methodologically, the use of transcription conventions is pivotal when presenting recorded spoken data in a written format for micro-level analysis of discourse. Particularly, the Jefferson transcription system is used by many discourse analysts and conversation analysts.^45^ Transcription conventions are used to capture the conversation as it occurs naturally, including, for example, speaker's overlaps ([words]), a brief pause (.), prolongation of an utterance (:::), and the transcriber's comment or nonverbal activity ((*italic*)). Detailed transcription helps researchers analyse beyond the literal meaning of transcribed texts and capture the nuanced meaning (i.e., situated meanings) of social interaction in its given context.

## Conclusions

Discourse analysis is a useful methodological approach for researching medical education, which affords several viewpoints, including the relationship between language and power in society (macro), discursive practice in a social context (meso), and discourse structure and function (micro). Particularly, discourse-oriented studies at micro-and meso-levels focus on contextual interaction, which entails looking beyond the literal meaning of language and relating it to the social, cultural, and psychological dimensions of communicative events. Thus, it can help us unpack the black box of the learning process facilitated through interactions in medical education. Detailed analyses of interactions can add novel perspectives on education practices and present not only new avenues for researchers' theoretical contributions but also practical pedagogical implications for the medical education field.

Discourse analysis might be challenging for medical educators. Echoing Woodward-Kron, Stevens, and Flynn,^46^ the methodology's interdisciplinary research approach necessitates collaboration between medical education researchers and social scientists to achieve a deeper method of analysing the language used in specific contexts. Moreover, some online interdisciplinary communities for discourse and communication studies, such as DiscourseNet,^47^ offer useful discourse analysis learning resources and training programmes.

The recent COVID-19 pandemic has driven the development of online education and training in synchronous and asynchronous settings, making researching online discourse increasingly important. Discourse analysis can be a key methodology to investigate this at macro-, meso-, and micro-levels.

### Acknowledgments

This work was supported by the JSPS KAKENHI Grant-in-Aid for Scientific Research (C) Number 20K10374.

### Conflict of Interest

The authors declare that they have no conflict of interest.
